# Progressive Aggregation of 16 kDa Gamma-Zein during Seed Maturation in Transgenic *Arabidopsis thaliana*

**DOI:** 10.3390/ijms222312671

**Published:** 2021-11-24

**Authors:** Elsa Arcalis, Davide Mainieri, Alessandro Vitale, Eva Stöger, Emanuela Pedrazzini

**Affiliations:** 1 Department of Applied Genetics and Cell Biology, Institute of Plant Biotechnology and Cell Biology, University of Natural Resources and Life Sciences, 1190 Wien, Austria; eva.stoger@boku.ac.at; 2 Istituto di Biologia e Biotecnologia Agraria, CNR, 20133 Milano, Italy; davide.mainieri@isafom.cnr.it (D.M.); vitale@ibba.cnr.it (A.V.)

**Keywords:** seed development, storage proteins, prolamins, zeins, protein body biogenesis, endoplasmic reticulum

## Abstract

Prolamins constitute a unique class of seed storage proteins, present only in grasses. In the lumen of the endoplasmic reticulum (ER), prolamins form large, insoluble heteropolymers termed protein bodies (PB). In transgenic Arabidopsis (*Arabidopsis thaliana)* leaves, the major maize (*Zea mays*) prolamin, 27 kDa γ-zein (27γz), assembles into insoluble disulfide-linked polymers, as in maize endosperm, forming homotypic PB. The 16 kDa γ-zein (16γz), evolved from 27γz, instead forms disulfide-bonded dispersed electron-dense threads that enlarge the ER lumen without assembling into PB. We have investigated whether the peculiar features of 16γz are also maintained during transgenic seed development. We show that 16γz progressively changes its electron microscopy appearance during transgenic Arabidopsis embryo maturation, from dispersed threads to PB-like, compact structures. In mature seeds, 16γz and 27γz PBs appear very similar. However, when mature embryos are treated with a reducing agent, 27γz is fully solubilized, as expected, whereas 16γz remains largely insoluble also in reducing conditions and drives insolubilization of the ER chaperone BiP. These results indicate that 16γz expressed in the absence of the other zein partners forms aggregates in a storage tissue, strongly supporting the view that 16γz behaves as the unassembled subunit of a large heteropolymer, the PB, and could have evolved successfully only following the emergence of the much more structurally self-sufficient 27γz.

## 1. Introduction

The polypeptides of seed storage proteins undergo different interchain interactions that are important for their synthesis, intracellular traffic, and accumulation. At the end of seed development and irrespective to their final subcellular compartment of accumulation—protein storage vacuoles (PSV) or protein bodies (PB) directly derived from the endoplasmic reticulum (ER)—storage proteins appear to the electron microscope as condensed electron-dense structures [1,2]. The origin and biochemical features of these structures are, however, quite variable, depending on the specific storage protein and the tissue of accumulation (endosperm or embryo). Trimerization of newly synthesized 7S/11S globulins, mainly due to hydrophobic interactions, is required for their approval by ER quality control and consequent intracellular traffic to PSV [3]. Further, transient polymerization of vacuolar storage proteins occurs during traffic and also has a role in correct sorting. This can be visualized both biochemically and by the detection of electron-dense structures in the Golgi cisternae or late endosomes en route to storage vacuoles [4,5,6]. In seeds of certain plants, such as pumpkin *(Cucurbita* sp cv Kurokawa Amakuri Nankin), transient polymerization occurs already in the ER lumen, leading to the formation of electron-dense precursor-accumulating (PAC) vesicles that reach storage vacuoles bypassing the Golgi apparatus [7]. All these transient, large structures that may involve the two major classes of vacuolar storage proteins—7S/11S globulins and the monomeric 2S albumins—are easily solubilized in aqueous buffers, whereas the major storage proteins of cereals—prolamins—form very large insoluble PBs in the ER [8]. In Panicoideae and rice (*Oryza sativa*), PB permanently accumulates in the ER, whereas in other cereals such as wheat they are delivered to storage vacuoles at late stages of seed maturation, bypassing the Golgi apparatus. A major feature of PBs permanently located in the ER is the presence of extensive interchain disulfide bonds that lead to protein insolubilization [9].

Zeins, the prolamins of maize (*Zea mays*), can be divided into four classes: alpha (more than thirty genes), gamma (three genes that produce polypeptides of 27, 50, and 16 kDa), beta (a single gene), and delta (two genes that encode for 10 kDa and 18 kDa polypeptides) [10,11]. Alpha and delta zeins constitute the core of the PB, whereas 27 kDa and 50 kDa gamma zeins (27γz, 50γz) form the outer PB layer, in contact with the luminal face of the ER membrane, and 16 kDa gamma zein (16γz) is at the interface between the inner core and outer PB layer [2,12]. 27γz, the single most abundant PB polypeptide, forms homotypic, insoluble, electron-dense PBs also when ectopically expressed in vegetative tissues [13,14]. Unlike α-zeins, which can be solubilized by aqueous alcohol, 27γz and 50γz are solubilized by reducing buffers; in agreement with this, progressive mutagenesis of the Cys residues involved in the interchain bonds of 27γz results in its increasing solubility and ability to traffic from the ER along the secretory pathway [15]. 27γz and 50γz have orthologs in other Panicoideae, but 16γz has instead been found only in maize and has originated, most probably from 27γz, as a consequence of the relatively recent whole-genome duplication of this cereal [16]. With respect to 27γz, 16γz has lost part of the Cys residues involved in interchain bonds and a large portion of a Pro-rich repeated domain [17]. Unlike its paralogous zein 27γz, 16γz is unable to form PBs when expressed ectopically in transgenic vegetative tissues, where instead it polymerizes into disordered electron-dense threads that markedly enlarge the ER lumen [14]. The protein is also partially soluble in aqueous buffers in the absence of reducing agents, but when co-expressed with 27γz it becomes fully insoluble unless reduced, supporting the hypothesis that in a natural PB 16γz interacts with 27γz and has the role of establishing ordered interactions between the inner PB core and the outer PB layer [14].

In this work, we have investigated whether the peculiar features of 16γz are also maintained when the protein is synthesized in a storage seed tissue, where other types of storage proteins are synthesized as well, and where a development program is active to favor their optimal accumulation.

## 2. Results

### 2.1. 16γzf Accumulates in Arabidopsis Embryos without Interfering with the Synthesis and Processing of Endogenous Storage Proteins

**Figure 1:**
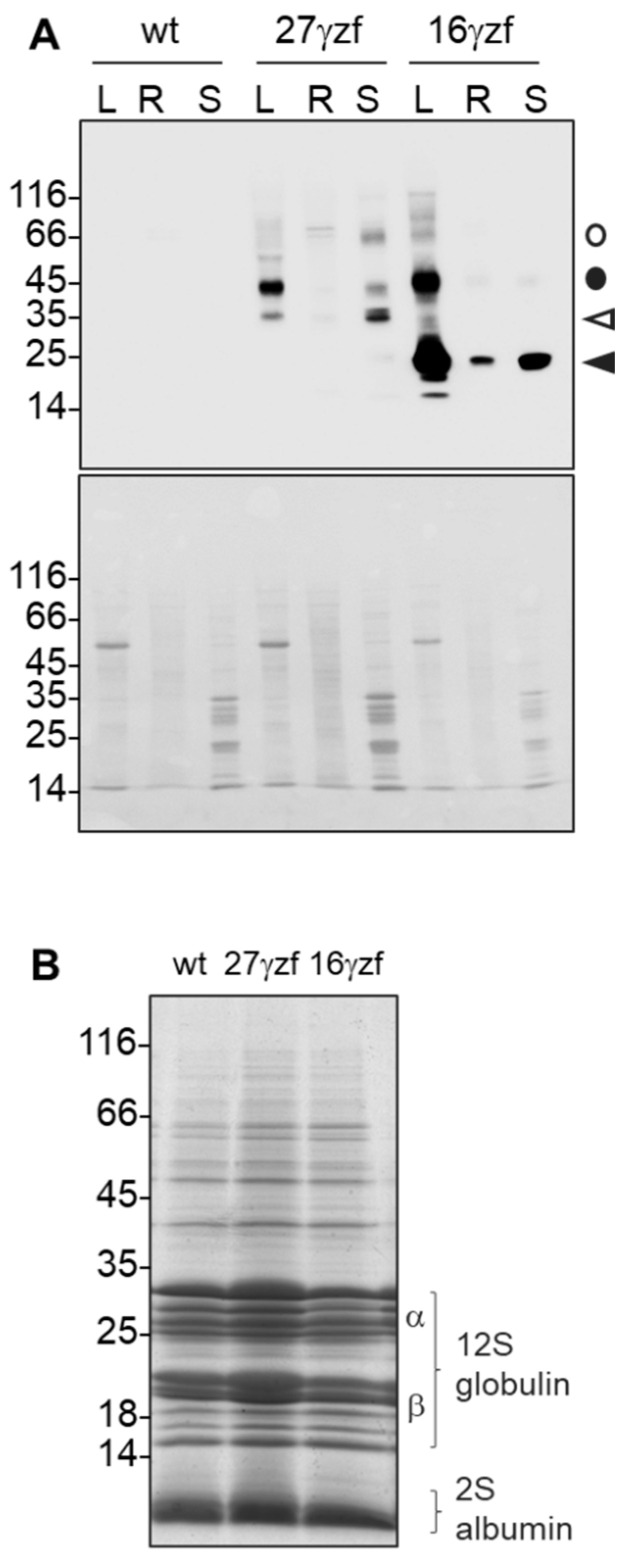
27γzf and 16γzf accumulate in dry seeds and do not interfere with the sorting of endogenous Arabidopsis storage proteins. (**A**): Total protein homogenates were prepared with denaturing buffer from leaves (L), roots (R), or dry seeds (S) of Arabidopsis constitutively expressing FLAG-tagged versions (f) of 27γz or 16γz, or from wild-type (wt) plants. Top panel: Analysis by SDS-PAGE and protein blot with anti-FLAG antibodies. The position of 27γzf (empty arrowhead), 16γzf (black arrowhead) monomers and their respective dimeric forms (empty and black circles, respectively) are indicated. Bottom panel: total proteins staining with Ponceau S. (**B**): Analysis of total protein extracts from dry seed by SDS-PAGE and Coomassie blue staining. The positions of the mature polypeptides of the endogenous 12S globulins and 2S albumins are indicated. Numbers on the left in both A and B indicate the position and size (kDa) of molecular mass markers.

Leaf, root, or seed proteins from Arabidopsis (*Arabidopsis thaliana*) transgenic plants singly expressing 27γz or 16γz C-terminal tagged with a FLAG epitope (27γzf and 16γzf, respectively [14]), were analyzed to compare the accumulation and electrophoretic patterns of the recombinant proteins in seeds and vegetative tissues. Proteins were extracted from dry seeds, or from leaves or roots of 6-week-old plants, with homogenation buffer supplemented with reducing agent. Protein blot with anti-FLAG antibodies showed that accumulation of both zeins was highest in leaves and lowest in roots (Figure 1A). Based on the known electrophoretic migration of the different forms of the two zeins [14], the relative amount of unmodified 27γzf monomers (Figure 1A, empty arrowhead) with respect to the modified form (migrating between the 35 and 45 kDa markers) was markedly higher in seeds than in vegetative tissues. Minor proportions of incompletely denatured dimers and larger polymers were also detected as lower migration forms (empty circle). In all tissues, 16γzf was detected mainly as monomers (black arrowhead) and in minor proportion, dimers (black circle), with no marked, qualitative tissue-specific differences. 

Overall, electrophoretic analysis indicates that both zeins expressed under the CaMV35S promoter accumulate in seeds to levels comparable to those in leaves. No fragmentation product still containing the FLAG epitope was detected. Since the modification of 27γz expressed in vegetative tissues most probably consists of hydroxylation of proline residues [13,15], the results suggest lower proline hydroxylation activity in seeds. Such hydroxylation seems to be mainly limited to vegetative tissues since proline residues of 27γz naturally synthesized in maize endosperm are also unmodified [18].

The major Arabidopsis seed storage proteins belong to the 12S and 2S classes; their precursor polypeptides (around 50 kDa and 15 kDa for 12S and 2S, respectively) are proteolytically matured only upon correct traffic from the ER and sorting to PSVs [19]. The SDS-PAGE patterns of total seed proteins did not show any change in the zein-expressing seeds compared to the wild-type plants (Figure 1B), indicating that the two zeins constitute only a very minor percentage of total proteins.

### 2.2. 16γzf Avoids Traffic to PSVs and Forms Electron-Dense Threads That Progressively Coalesce during Embryo Development

**Figure 2:**
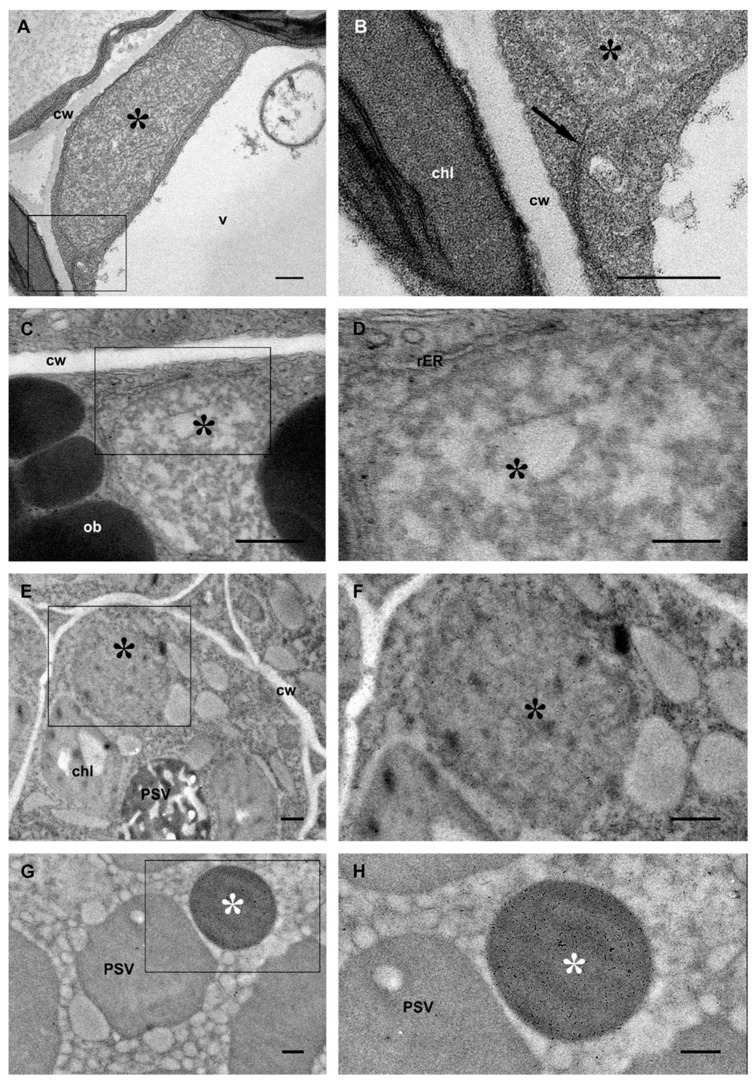
Progressive coalescence of 16γzf during embryo development. (**A**,**B**): leaves; (**C–F**): developing embryos (11 dap); (**G**,**H**): mature embryos. (**A–D**): Ultrathin sections post-fixed with osmium. (**E**–**H**): Immunolabelling with anti-FLAG antibody and secondary 10 nm gold-conjugated donkey anti-rabbit serum. Boxes in (**A,C,E,G**) indicate the regions that are shown at higher magnification in (**B**,**D**,**F**,**H**), respectively. Symbols and acronyms: 16γzf threads and loosely packed structures (black asterisks), ER membrane continuous with the membrane enclosing the 16γzf threads (arrow), homogeneously electron-dense structures (white asterisks), cell wall (cw), chloroplast (chl), oil bodies (ob), protein storage vacuoles (PSV), and vacuole (v). Scale bars in (**A,C,E,G**) and (**B,D,F,H**) are 1 µm and 0.5 µm, respectively.

16γzf is unable to form PBs in Arabidopsis transgenic leaves, but accumulates as electron-dense thread-like structures that enlarged the ER lumen [14] (Figure 2A,B). To verify if these structures are also formed in seeds, the subcellular localization and morphology of 16γzf during seed development was determined by immunogold electron microscopy (EM) with anti-FLAG antibodies.

Mature embryos showed labeling of 16γzf in round structures with diameters of about 1 µm and nearly homogeneous electron-dense content (Figure 2G and magnification in Figure 2H, white asterisk), resembling homotypic PBs formed by 27γzf in leaves [14], and markedly different from the 16γzf threads that irregularly enlarge the leaf ER lumen (conventional EM analysis in Figure 2A and magnification in Figure 2B, see also Figure 7 in [14]). In immature embryos, conventional (Figure 2C and magnification in Figure 2D) and immunogold (Figure 2E and magnification in Figure 2F) EM analysis showed structures having an intermediate pattern between those of leaves and mature embryos: 16γzf accumulated in smaller (around 2 µm) and less irregularly shaped compartments than those in leaves, but not as round-shaped and homogeneously electro-opaque as those in the mature embryos (in Figure 2 compare panels C–F black asterisks, with panels G,H white asterisks). In immature embryos, 16γzf was organized in threads that appeared more dispersed in the compartment lumen than those in leaf cells. PSVs, where Arabidopsis storage proteins accumulate, were clearly devoid of 16γzf (PSV in Figure 2G,H). In immature embryos, the PSVs content appeared very dissimilar in electron density and shape from those of 16γzf structures (compare PSV and asterisks in Figure 2E).

### 2.3. 16γzf Forms Insoluble Aggregates in Mature Embryos, Unlike 27γzf

**Figure 3:**
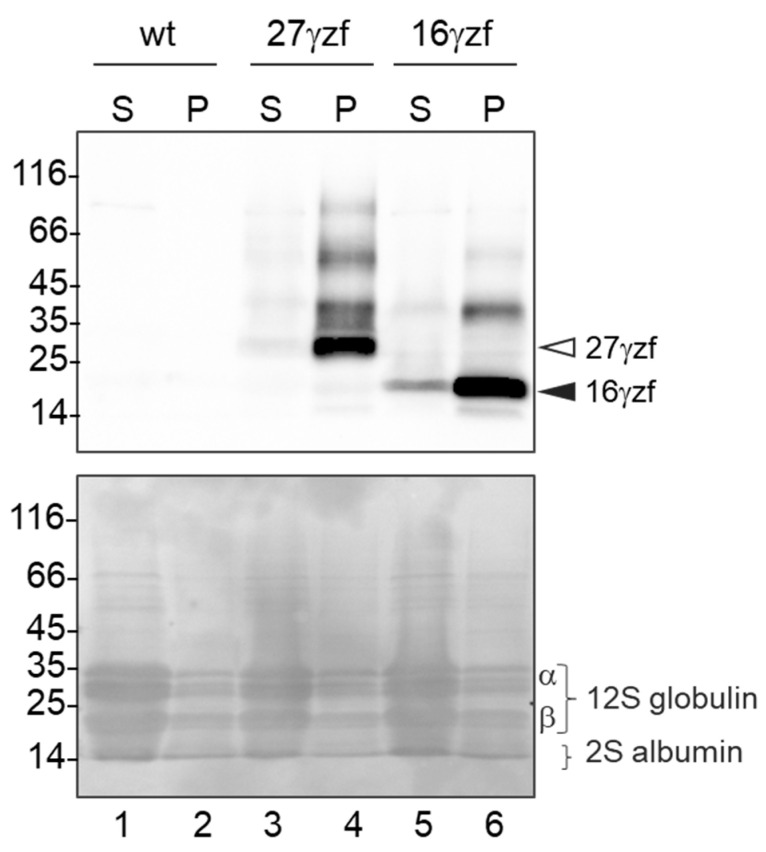
16γzf PB-like structures visualized by EM are mainly insoluble in salt buffer, similar to 27γzf PBs. Dry seeds from wt, 27γzf, or 16γzf plants were ground and homogenized in saline buffer without reducing agents or detergent. Soluble (S) and insoluble (P) fractions were separated by centrifugation. Both (S) and (P) fractions were analyzed by SDS-PAGE followed by protein blot. Top panel: anti-FLAG antibodies. The positions of 27γzf and 16γzf monomers are indicated by the empty and the black arrowheads, respectively. Bottom panel: Ponceau S total protein staining. The positions of the Arabidopsis endogenous seed storage proteins 12S globulins (α and β subunits) and 2S albumins are indicated. Numbers on the left indicate the position and size (kDa) of molecular mass markers.

Based on the morphology of the structures formed by 16γzf in leaves, its PB-like condensation in mature seeds was unexpected. It was therefore determined whether the biochemical features of 16γzf accumulated in mature seeds were also similar to those of PB-forming 27γzf. Dry seeds were first homogenized in saline buffer without detergent and subjected to low-speed centrifugation. Both zeins were recovered in the pellet fractions, with marginal amounts of 27γzf and only a slightly higher proportion of 16γzf remaining in the supernatant (Figure 3). Therefore, the 16γzf PB-like structures visualized by EM are largely insoluble in salt buffer, similarly to 27γzf PBs.

**Figure 4:**
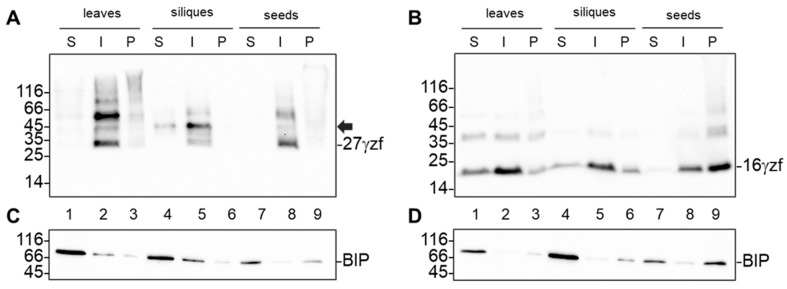
16γzf forms insoluble aggregates in mature embryos, unlike 27γzf. Leaves, immature embryos (siliques) or dry seeds from 27γzf (**A**,**C**) or 16γzf (**B**,**D**) transgenic Arabidopsis plants were homogenized in saline buffer supplemented with nonionic detergent to solubilize membranes, in the absence of reducing agents. Soluble (S) proteins were collected after centrifugation. Pellets were resuspended in the same buffer supplemented with 4% 2-Mercaptoethanol to reduce disulfide bonds; the new soluble proteins (I) were separated from the completely insoluble pellet (P) by centrifugation. Analysis was done by SDS-PAGE and protein blot. (**A**,**B**): anti-FLAG antibodies; the positions of 27γzf and 16γzf monomers are indicated. The black arrow in A indicates the 27γzf modified form only present in vegetative tissues. (**C**,**D**): anti-BIP antibodies; the position of BiP is indicated.

We then compared the solubility of the two zeins in leaves and in immature or mature seeds by sequential extraction in different buffers. In all the three tissues analyzed, 27γzf was almost completely insoluble unless reduced (Figure 4A, lanes 2, 5, 8), a feature of natural 27γz accumulated in maize endosperm PBs and already observed in transgenic leaves [14]. In leaves, and to a minor extent in developing seeds, 16γzf was also partially soluble in the absence of a reducing agent (Figure 4B, lanes 1, 4). No soluble polypeptides were, however, detected in dry seeds, where the major proportions of 16γzf could not be solubilized even by reducing agent, indicating that a large part of the morphologically PB-like 16γzf structures is not held together by interchain disulfide bonds, but is actually due to aggregation (Figure 4B, lane 9).

The major ER chaperone BiP has affinity to not yet folded and assembled polypeptides and to misfolded proteins [20,21]. In transgenic Arabidopsis leaves, BiP1/2 (the two highly homologous, major Arabidopsis BiP polypeptides, which cannot be distinguished from one another by antibodies) are found associated with both gamma zeins, but in higher proportion to 16γzf, most probably because the N-terminal domain of this zein has high affinity to BiP and because its assembly in the absence of other zein partner polypeptides could remain permanently unresolved [22]. Protein blot with anti-BiP antiserum showed that, in 27γzf leaves and immature embryos, even if the most of polypeptides were in the S fraction (BiP is per se a soluble protein), a minor but not irrelevant proportion of BiP1/2 was solubilized only after reduction (Figure 4C, lanes 2, 5), probably because associated to newly synthesized, not yet polymerized 27γzf polypeptides. In dry seeds, where protein synthesis is terminated, no reduced-soluble BiP was detected (Figure 4C, lane 8). The solubility of BiP1/2 was quite different in the tissues expressing 16γzf. There was almost no reduced-soluble BiP1/2 in any tissue, whereas fully insoluble BiP1/2 increased during seeds maturation, reaching about half of the total in dry seeds (Figure 4D, lanes 3, 6, 9). This paralleled the increasing full insolubilization of 16γzf, strongly suggesting extended interactions between this zein and the chaperone also during embryo development, which persist in mature embryos. 

## 3. Discussion

The results reported here show that electron-dense 16γzf threads, similar to those accumulated in the ER of Arabidopsis leaf cells, are present in developing embryos and that these then coalesce into smaller, nearly homogeneously electron-dense structures that accumulate in mature seeds. These structures are regularly round-shaped and have diameters up to 1 µm, thus morphologically resembling the homomeric PBs formed by 27γzf in Arabidopsis leaves [14]. However, the results also show that most 16γzf present in mature, dry seeds is also insoluble in the presence of reducing agents that fully solubilize seed-accumulated 27γzf, as well as both recombinant zeins accumulated in leaf cells [14]. This indicates that 16γzf coalescencing into morphologically PB-like structures is not a process of ordered polymerization; rather, it reflects most probably unregulated hydrophobic interactions leading to aggregation. The lack of detectable precursors of endogenous storage proteins in the transgenic seeds indicates that neither 16γzf nor 27γzf, at least at the levels of synthesis allowed by the 35S promoter, interfere with the traffic, processing, and correct intracellular sorting of 12S and 2S polypeptides to PSVs.

Seed storage proteins evolved to maximize the protein-to-volume of storage compartment ratio; polypeptides are therefore highly packed, but they avoid falling into an aggregate state that can compromise their hydrolysis during germination. Electron-dense accretions of soluble seed storage proteins can form in the ER naturally or in transgenic plants. In pumpkin [7] and castor bean (*Ricinus communis*) [23] seeds, similar accretions containing 2S albumins and 11S globulins occur naturally, being then concentrated into PAC vesicles and then transported to PSV. These are correctly assembled and folded polypeptides that do not permanently aggregate into insoluble structures [7,23]. In soybean (*Glycine max*), suppression of two members of the 7S globulin gene family (conglycinins) induces the formation of PB-like structures in the ER, that entrap the precursor forms of other conglycinins as well as additional PSV polypeptides [24]. Biochemical analysis showed that these precursors were assembled into soluble trimers, but cannot proceed along the pathway to PSVs. Sunflower (*Helianthus annuus* L.) 2S albumins [25] or pea (*Pisum sativum*) 7S globulin [26] with an added C-terminal sequence for retention in the ER, can form in transgenic plants spherical electron-dense structures, which are solubilized in a saline buffer. All these unusual formations of PB-like structures in the ER are therefore condensations of correctly folded polypeptides rather than aggregations of misfolded polypeptides. Normally soluble, secreted proteins can also change their fate when ectopically expressed in storage tissues: naturally secreted phytase is included into zein PBs at late stages of maturation, indicating that PBs can be a dominant destination in the secretory pathway [27]. However, extraction with saline buffer in reducing conditions solubilized the recombinant phytase.

The aggregation of 16γzf is therefore a specific process, unrelated to the other above described examples of PB-like structure formations. The peculiarity of the process is also indicated by the unusual, progressive full insolubilization of BiP that parallels 16γzf insolubilization during embryo maturation. This is not observed in embryos expressing the PB-forming 27γzf and indicates that the extended interactions between 16γzf and the chaperone observed in Arabidopsis leaves [22] also occur during embryo development and persist in the mature embryo.

16γz’s position and intermediate solubility between those of α-zeins and the two other γ-zeins in natural maize PBs, its very unique polymerization into threads, high affinity to BiP, and high stimulation of the unfolded protein response when expressed individually in transgenic leaves are consistent with the hypothesis that 16γz evolved as a contact structure between the outer layer and the inner core of maize PBs [2,12,14,22]. The acquisition of a stable conformation would thus remain unaccomplished in the absence of the other partner zeins. The results presented here provide additional support to this scenario. During seed desiccation, late embryogenesis abundant (LEA) proteins and a number of small heat shock proteins (sHSP) are specifically synthesized and are believed to avoid damages, including irreversible protein aggregation, caused by the decrease of water availability [28,29,30]. Certain LEA and sHSP are located in the ER and thus may be involved in avoiding storage protein aggregation that would hamper their hydrolysis during germination. In vitro experiments indicate that, at a low ratio to the substrate, the anti-aggregation function of a model LEA is impaired [31]. As seeds progress to desiccation, it is thus possible that these aggregation inhibitors, as well as the ubiquitous chaperones like BiP, become progressively unable to inhibit unspecific interactions between 16γzf polypeptides, which would thus start to form fully insoluble, irreversible aggregates that cannot be solubilized even by treatments that reduce interchain disulfide bonds. This strengthens the view that 16γz behaves as the unassembled subunit of a large heteropolymer, the PB, and could have evolved successfully only following the emergence of the much more structurally self-sufficient 27γz [14,22].

ER quality control exerts three functions in the secretory pathway: it favors fold-ing and assembly of newly synthesized proteins, avoids their traffic until a native state has been achieved and targets for degradation polypeptides that fail for too long time to become competent for traffic. The ability to take advantage of the first two activities of quality control and at the same time avoid the third one is a major, unique feature of prolamins and PB assembly. In this respect, it is remarkable that even 16γz does not seem to undergo marked long term degradation under the stressing conditions of seed desiccation and does not disturb the normal structural maturation and traffic of PSV endogenous storage proteins, in spite of the fact that it very strongly challenges ER quality control compared to 27γz and, unlike the latter, is unable to form a typical PB in the absence of the other zein partners. Besides making 16γz a useful model to study the molecular features that regulate the balancing between folding, degradation, and aggregation in the ER, this may also be relevant for possible strategies to improve the protein nutritional quality of seeds through protein engineering strategies.

## 4. Materials and Methods

### 4.1. Plant Material

Transgenic Arabidopsis plants (ecotype Columbia) expressing 27 kDa γ-zein or 16 kDa γ-zein C-terminally tagged with the FLAG epitope DYKDADDDK—and named 27γzf and 16γzf, respectively [22]—or wild-type plants were grown in sterile conditions on half-concentrated Murashige and Skoog solid media (Duchefa Biochemie), 10 g/L Sucrose, 0.8% (*w*/*v*) phyto agar (Duchefa Biochemie) at 23 °C under a 16/8 h light/dark cycle.

Siliques were harvested on the eighth day after flowering.

### 4.2. Antibodies

The following antisera or antibodies were used, at the indicated dilutions. Rabbit polyclonal anti-FLAG (1:2,000 Sigma Aldrich, St. Louis, MO, USA); rabbit polyclonal anti-tobacco (*Nicotiana tabacum*) BIP (1:10,000 [3]); goat anti-rabbit IgG-peroxidase conjugate (1:16,000, Pierce Biotechnology Rockford, IL, USA).

### 4.3. Total Protein Extraction from Leaves, Roots, and Dry Seeds

Leaves or roots, 2–3 cm in length from 6-week-old plants were ground in liquid nitrogen and homogenized with five volumes (5:1 *V/W*) of buffer T (150 mM NaCl, 1.5 mM EDTA, 1.5% Triton X-100, 150 mM Tris-Cl pH 7.5) supplemented with cOmplete™ Protease Inhibitor Cocktail (Life Technologies, Wien, Austria). Dry seeds were homogenized using the same procedure, but with a 25:1 buffer volume/weight ratio.

Equal amounts of total proteins from each sample were denatured and loaded on 15% SDS-PAGE followed by protein blot incubation with the appropriate antiserum or antibody, and detection with SuperSignal West Pico Chemiluminescent substrate (Thermo Scientific, Rockford, IL, USA). Unstained Protein MW Markers (Fermentas, Vilnius, Lithuania) were used as molecular mass markers. Total proteins were stained with Ponceau S, for normalization and as a loading control.

### 4.4. Protein Body Precipitation

Dry seeds were ground in liquid nitrogen and homogenized with eight volumes (8:1 V/W) of buffer A (10 mM KCl, 2 mM MgCl_2_, 100 mM Tris-Cl pH 7.8) supplemented with cOmplete™ Protease Inhibitor Cocktail (Life Technologies). The homogenates were centrifuged at 1500× *g* for 10 min at 4 °C. The supernatants were saved (soluble fraction, S). The pellets, containing protein bodies, were resuspended in the same volume of buffer A supplemented with 4% 2-mercaptoethanol (2-ME) and incubated for 30 min at room temperature to reduce the disulfide bonds (insoluble proteins unless reduced, I). Equal volumes of soluble (S) and insoluble (I) fractions were then adjusted to 1.0% SDS, 4% 2-ME, and analyzed by 15% SDS-PAGE followed by protein blot incubation as described above.

### 4.5. Protein Sequential Extraction with Different Buffer and Centrifugation

Dry seeds, 8-day-old siliques, or 2–3 cm long leaves were ground in liquid nitrogen and homogenized with fifteen volumes (15:1 *V/W*) of buffer T (150 mM NaCl, 1.5 mM EDTA, 1.5% Triton X-100, 150 mM Tris-Cl pH 7.5) supplemented with cOmplete™ Protease Inhibitor Cocktail (Life Technologies). After 30 min of incubation in ice, the homogenates were centrifuged at 1500× *g* for 10 min at 4 °C. The supernatants were saved (soluble fraction, S). The pellets were resuspended in the same volume of buffer T supplemented with 4% 2-mercaptoethanol (2-ME), incubated for 30 min at 4°C to reduce the disulfide bonds, and centrifuged at 1500× *g* for 10 min at 4 °C. The recovered supernatant contained proteins insoluble unless reduced (I). The pellets contained fully insoluble material (P). Soluble (S), insoluble (I), and pellet (P) fractions were then adjusted to 1.0% SDS, 4% 2-ME, and analyzed by 15% SDS-PAGE followed by protein blot incubation as described above.

### 4.6. Electron Microscopy

For immunolocalization, transgenic isolated mature embryos, as well as developing embryos (bent cotyledon) expressing 16γzf were fixed and processed as described before [27]. Shortly, embryos were fixed in 4% paraformaldehyde and 0.5% glutaraldehyde in 0.1 M cacodylate buffer pH 7.4 for 2h at room temperature. After several washes with 0.1 M cacodylate buffer, samples were dehydrated through ethanol series and then infiltrated with LRWhite resin. Immunolocalization was performed with rabbit anti-FLAG antibodies on sections showing silver interferences collected on copper grids as previously described [27]. The antigen–antibody reaction was visualized with donkey anti-rabbit serum labeled with 10 nm gold particles. Imaging was performed under a FEI Tecnai G2 electron microscope. 

For ultrastructural analysis, sections obtained from leaves expressing 16γzf were fixed as described [27]. Briefly, samples were fixed in 2% paraformaldehyde and 2.5% glutaraldehyde in 0.1M cacodylate buffer pH 7.4 overnight at 4 °C. After several washes in 0.1 M cacodylate buffer pH 7.4, leaf sections were subjected to double osmium impregnation, followed by en bloc staining with uranyl acetate and lead aspartate. Leaf samples were dehydrated through ethanol series and progressively infiltrated and embedded with low viscosity epoxy resin. Thin sections showing silver interferences were mounted on copper grids prior to imaging. Developing embryos (bent cotyledon) expressing 16γzf were isolated and fixed and processed as described in [32] with slight modifications. Thus, embryos were fixed in 2% paraformaldehyde and 2.5% glutaraldehyde in 0.1 M sodium cacodylate buffer, pH 7.4 O/N at 4 °C. After several washes with 0.1 M sodium cacodylate buffer, pH 7.4, samples were incubated in 1% (*w*/*v*) tannic acid in 0.1 M Cacodylate buffer, followed by 1% (*w*/*v*) aqueous osmium tetroxide. Subsequently, embryos were dehydrated through ethanol series and embedded in low viscosity epoxy resin. Sections showing silver interferences were collected on copper grids and imaged using a FEI Tecnai G2 transmission electron microscope operating at 160 kV.

## Data Availability

Not applicable.
